# Metal Toxicity across Different Thallus Sections of the Green Macroalga, *Ulva australis*

**DOI:** 10.3390/toxics11070548

**Published:** 2023-06-22

**Authors:** Hojun Lee, Geonhee Kim, Stephen Depuydt, Kisik Shin, Taejun Han, Jihae Park

**Affiliations:** 1 Bio Environmental Science and Technology (BEST) Lab, Ghent University Global Campus, 119-5, Songdomunhwa-ro, Incheon 21985, Republic of Korea; hojun.lee@ugent.be (H.L.); taejun.han@ghent.ac.kr (T.H.); 2 Department of Marine Science, Incheon National University, 119, Academy-ro, Yeonsu-gu, Incheon 22012, Republic of Korea; 3 Erasmus Brussels University of Applied Sciences and Arts, Nijverheidskaai 170, 1070 Brussels, Belgium; stephen.depuydt@ehb.be; 4 Water Environmental Engineering Research Division, National Institute of Environmental Research (NIER), 42, Hwangyeong-ro, Incheon 22689, Republic of Korea; envi95@korea.kr; 5 Department of Animal Sciences and Aquatic Ecology, Ghent University, Coupure Links 653-Block F, B-9000 Gent, Belgium; 6 Centre for Environmental and Energy Research, Ghent University Global Campus, 119-5, Songdomunhwa-ro, Incheon 21985, Republic of Korea

**Keywords:** bioassay, metals, multi-endpoints, thallus differentiation, risk assessment

## Abstract

We aimed to identify functional differences between different sections of the thallus of *Ulva australis* and develop tissue-endpoint combinations to assess the toxicity of six metals (i.e., Ag, As, Cd, Cr, Cu, and Ni). EC_50_ values for these metals in three sections of the thallus of *Ulva* were obtained for multiple endpoints: relative growth rate (RGR), chlorophyll *a* fluorescence, pigment contents, and the expression of the photosynthesis-related gene, *rbcL*. The responses of the endpoints varied across the respective thallus sections; overall, the most toxic metals were Ag and Cu. These endpoints were the best for evaluating metal toxicity: ETR_max_ of the middle thallus sections for Ag toxicity; RGR of the middle thallus section for As and Cd; ETR_max_ of the marginal thallus section for Cr; Chl *b* contents of the marginal thallus section for Cu; RGR of the basal thallus section for Ni. The EC_50_ values for the inhibition of ETR_max_ in middle (0.06 mg∙L^−1^) and Chl *b* in the marginal thallus sections (0.06 mg∙L^−1^) were all lower than those of the quality standard for wastewater discharge values of Ag and Cu in Republic of Korea and the US, pointing to the suitability of *U. australis*-based endpoints for risk assessment.

## 1. Introduction

*Ulva australis* (i.e., sea lettuce) is a green seaweed species found in temperate and subtropical coastal waters worldwide. 

*U. australis* is morphologically simple; specifically, it has a flat, lettuce-like body that consists of two thick cell layers [1,2,3,4,5]. Overall, this green alga is thought to have a homogeneous thallus that has little functional differentiation.

However, Hiraoka and Enomoto [6] found that experimental disks excised from the thallus of *U. australis* showed different degrees of zooid formation depending on the part of the body from which the disk had been extracted. Han et al. [1] compared the morphology, pigmentation, photosynthesis, growth, reproduction, and UV-B sensitivity of *U. australis* across different thallus sections. The findings of this study were used to support the occurrence of adaptive functional differentiation among cells within the thallus. This differentiation was characterized as follows: the margins of the thallus were linked to high productivity and reproduction; the middle sections were linked to rapid growth; the basal sections were associated with the regeneration of new blade cells. This differentiation is thought to facilitate rapid adaptive responses when algae are subjected to unfavourable conditions [7]. However, to date, the morphological, physiological, and biochemical differences of the various sections (i.e., specifically, their responses to stressors other than UV radiation) of the thallus of *U. australis* have not been studied comprehensively.

Industrial and agricultural processes generate large amounts of wastewater; some of this water is then discharged into marine ecosystems [8]. Wastewater may contain various metals such as silver, arsenic, chromium, cadmium, copper, and nickel [9]. These metals are nonbiodegradable, toxic to non-target organisms, and can bioaccumulate via the food chain, posing a threat to the ecological integrity of oceans and human health [10]. In addition, it has been shown that exposure to high concentrations of metal can inhibit the growth, photosynthesis, and reproduction of algae. Under these conditions, algae also exhibit a heightened production of reactive oxygen species (ROS), which results in the cell membrane being damaged. This widespread and metal-exposure-driven DNA damage culminates in the development of metabolic disorders, which then eventually results in the death of the organism [11,12,13,14,15,16]. Therefore, it is necessary to create effective monitoring and management strategies, especially within the context of maintaining the ecological integrity of marine ecosystems in the ongoing Anthropocene. However, for such solutions to be developed, not only will metal pollution have to be quantified in marine ecosystems, it will have to be shown to pose a threat to marine life. 

As the detection of ecologically toxic substances and the assessment of their impact on biota are becoming increasingly important across the globe, various biomarker-based methods have been proposed and implemented. Unlike approaches that entail studying contaminants in isolation, this approach directly reflects the ecological dynamics associated with the contaminants of interest; additionally, it is cheaper and easier to apply than conventional chemical analyses. Specifically, aquatic bioassays provide a basis for assessing and managing the ecological risk that contaminants, including unknown substances, may pose to the aquatic environment. Specifically, multiple endpoint bioassays provide a more comprehensive risk assessment than single endpoint tests because they can effectively detect perturbations of specific contaminants, providing important insights into the mechanisms underlying the relative sensitivity of the measured endpoints [17,18].

The green macroalga, *U. australis* (i.e., formerly *Ulva pertusa*), inhabits shallow coastal waters and provides habitat and shelter for fish and invertebrates [2,19,20]. As an important primary producer in the food chain, the disturbance of *U. australis* populations may greatly affect the overall balance of many marine ecosystems across the globe. 

*U. australis* is an ideal model organism for ecotoxicity tests; for example, this species has been used to develop a test that has been applied to 75 different environmental samples containing contaminants such as metals, volatile organic compounds, herbicides, oils, dispersants, and slag waste [16,21]. The test measures the inhibition of the reproduction on the margins of the thallus by quantifying the colour changes exhibited by the thallus (i.e., caused by the release of reproductive cells) [20]. Ecotoxicity testing using *U. australis* has some advantages over conventional chemical techniques, namely, ease of use, sensitivity, cost, efficiency, environmental relevance, repeatability, and reproducibility [16,19,20,22,23,24,25,26].

In this study, we aimed to firstly investigate the functional differences among different sections of the thallus of *Ulva australis*. To achieve this aim, we evaluated the morphological, physiological, and biochemical characteristics of the different sections of the thallus. We then evaluated the sensitivity of each thallus section to six metallic toxicants (i.e., Ag, As, Cd, Cr, Cu, and Ni) to determine their respective suitability for toxicity testing. For each section, we measured various endpoints, including chlorophyll fluorescence, pigment concentration, growth, and *rbcL* expression. Overall, we elucidated specific thallus sections and endpoints that would be ideal for the toxicity testing of specific metals.

## 2. Materials and Methods

### 2.1. Algal Culture

*Ulva australis* samples were collected from sites near Ilgwang-myeon, Gijang-gun, Busan, Korea (35.283858° N, 129.259706° E). The collected samples were then stored in an artificial seawater medium at 15 °C under a white fluorescent light (FL20SS, Royal Philips, Amsterdam, The Netherlands). The medium was prepared by dissolving commercial sea salts (Coralife, Energy Savers Unlimited, Inc., Carson, CA, USA) and nutrients (1 mM KNO_3_ and 0.1 mM K_2_HPO_4_) in distilled water up to a concentration of 35 g∙kg^−1^.

### 2.2. Morphological Characteristics Comparisons

Algal disks (Ø 8 mm) were cut from different sections (i.e., marginal, middle, and basal sections) of the thallus of *U. australis*, and microscopic measurements for thallus thickness (in cross-section), cell size (random count in surface view), and number of cells [in a randomly selected 50 µm × 50 µm (2500 µm^2^) square] were determined (*n* = 8).

### 2.3. Growth

Growth was evaluated by measuring the size of the *U. australis* disks using an image analyser Moticam (Moticam 2.0 MP, Motic, Kowloon Bay, Hong Kong), and the relative growth rate (RGR) was calculated as follows:

RGR (% d^−1^) = [(ln(*A*_f_) − ln(*A*_i_))/*t*_f_)] × 100 (1)

where *A*_i_ and *A*_f_ are the initial and final disk areas and *t*_f_ is the test duration.

### 2.4. Chlorophyll a (Chl a) Fluorescence Measurements

Chl *a* fluorescence was measured using Imaging PAM (Heinz Walz GmbH, Effeltrich, Forchheim, Germany). After 72 h of treatment, the *U. australis* samples were dark-adapted for 15 min. Light pulses (0.15 µmol photons m^−2^∙s^−1^) from light-emitting diodes (LEDs) were then used to determine the initial fluorescence yield (*F*_o_), which indicates the fluorescence yield when all reaction centres of photosystem II (PSII) are open and plastoquinone A (Q_A_) is completely oxidized. Subsequently, a saturation pulse of approximately 5000 µmol photons m^−2^s^−1^ was applied to produce the maximum fluorescence yield (*F*_m_), induced by a short saturating pulse of actinic light, reducing all Q_A_ molecules. The value of *F*_v_/*F*_m_ was then calculated as follows:

*F*_v_/*F*_m_ = (*F*_m_ − *F*_o_)/*F*_m_ (2)

where *F*_v_ is the variable fluorescence.

Rapid light curves were then generated using 10 s pulses of actinic light gradually increasing from 0 to 1517 μmol photons m^−2^s^−1^. The maximum electron transport rate (ETR_max_) was calculated using the hyperbolic tangent equation given by Jassby and Platt [27]:

ETR = ETR_max_ × tanh(α × *I*/ETR_max_) (3)

where α and *I* denote ETR and irradiance, respectively, under light-limiting conditions.

Non-photochemical quenching (NPQ) was estimated using the following equation [28].

NPQ = (*F_m_* − *F*′*_m_*)/*F*′*_m_* (4)

where *F*′*_m_* denotes the maximum fluorescence yield observed under light-adapted conditions.

### 2.5. Pigment Contents 

Chlorophyll *a*, chlorophyll *b* (Chl *a* and Chl *b*), and carotenoids were extracted from *U. australis* disks in 1 mL methanol (≥99.9%; Sigma–Aldrich, St. Louis, MO, USA) for 24 h in the dark at 4 °C. The absorbance of the methanol extract was measured at 666 nm (Chl *a*), 653 nm (Chl *b*), and 470 nm (carotenoids) using a spectrophotometer (Scinco S-3100 PDA UV-Vis, Scinco, Seoul, Republic of Korea). The concentrations of Chl *a*, Chl *b*, and carotenoids were estimated using the equations reported by Lichtenthaler [29].

Chl *a* = 15.65 × A_666_ − 7.34 × A_653_ (5)

Chl *b* = 27.05 × A_653_ − 11.21 × A_666_ (6)

Carotenoids (Car) = (1000 × A_470_ − 2.86 × Chl *a* − 129.2 × Chl *b*)/245 (7)

where *A*_470_, *A*_653,_ and *A*_666_ represent the absorbances at 470 nm, 653 nm, and 666 nm, respectively. 

### 2.6. Metal Toxicity Testing 

Algal disks (Ø 8 mm) were cut from different sections of the healthy thallus (i.e., marginal, middle, and basal sections) and placed in 250 mL flasks with growth medium and different concentrations of the selected metals. Metal stock solution (Junsei, Tokyo, Japan) in deionized water was acidified following the addition of 1 N hydrochloric acid (HCl) or 1 M nitric acid (HNO_3_) to each flask to obtain the specified final concentrations (Table 1). The pH of all test solutions, including the control, was adjusted to a pH range of 8.0 ± 0.2 using 1 M NaOH and 1 M HCl solutions. The concentrations of carrier solvents used to facilitate the dissolution of metal contaminants have not been shown to cause or intensify toxicity in *Ulva*. Controls consisted of artificial seawater medium without toxicants. During incubation, air bubbles were gently integrated into the medium. Thallus disks were grown for 72 h under optimal environmental conditions (i.e., photon irradiance, 100 µmol photons m^−2^ s^−1^ of white, fluorescent light with 12:12 h L:D photoperiod, salinity 30 g∙kg^−1^, and temperature 15 ± 1 °C), the disks treated with the metals were subsequently removed, and their growth, chlorophyll fluorescence, and pigment content were quantified.

### 2.7. Gene Expression (qRT-PCR)

For gene expression analysis, silver (Ag) and copper (Cu) were selected due to their high toxicity across all examined endpoints. Additionally, the exposure concentrations used in this experiment were chosen by selecting the EC_50_ values from among the most sensitive and reliable endpoints.

After a 72-h exposure to 0.12 mg∙L^−1^ of Ag and Cu, *U. australis* disks from different thallus sections were harvested and ground in liquid nitrogen. Total RNA was extracted from each sample using an RNeasy Plant Mini Kit (Qiagen, Hilden, Germany), and cDNA was prepared from 1 µg of total RNA using a Diastar RT Kit (SolGent Co., Ltd., Daejeon, Republic of Korea) according to the manufacturer’s instructions. The purity and concentrations of RNA and cDNA were determined using a Nanodrop UV spectrophotometer (Thermo Fisher Scientific, Waltham, MA, USA). 

qRT-PCR was performed using a 2×RT PCR Smart Mix (with SYBR Green) (SolGent Co., Ltd., Daejeon, Republic of Korea) on a CFX Connect Real-Time PCR Detection System (Bio-Rad, Hercules, CA, USA). Amplification was performed through denaturation at 95 °C for 5 min, followed by 40 cycles of 94 °C for 30 s, 57 °C for 30 s, and 70 °C for 10 s. The primers for the reference gene (18S rRNA) and the tested gene (*rbcL*) were as follows: 5′ -CACGTCTTGGTGAATCATGG-3′ (*18S rRNA*-forward), 5′-CTTGGATGTGGTAGCCGTTT-3′ (*18S rRNA*-reverse), 5′- AGAAATGATGGAGCGTGGTC-3′ (*rbcL*-forward), 5′-TGGTCACCACCTGACATACG-3′ (*rbcL* -reverse).

### 2.8. Statistical Analyses 

One-way analysis of variance (ANOVA) followed by multiple least significant difference (LSD) comparison tests were performed to test for differences between the endpoints and the sections. Results were presented as half maximal effective concentration (EC_50_) values with 95% confidence intervals (95% CI) and estimated using linear interpolation methods (Toxical 5.0, Tidepool Scientific Software, McKinleyville, CA, USA). The accuracy of the test was estimated by calculating the coefficient of variation (CV), which is the standard deviation expressed as a percentage of the mean.

The sensitivity and reliability of the six endpoints for metals were compared based on their EC_50_ and CV values, respectively. The EC_50_ and CV values of all endpoints for each metal were ranked in descending order regardless of the specific thallus sections; then, scores from 1 to 18 (six endpoints and three sections) were assigned to the two sequences. Mean scores were assigned to the same sequence. A lower score indicates a more sensitive and reliable endpoint.

## 3. Results and Discussion

### 3.1. Morphological, Physiological, and Biochemical Traits by Thallus Section

**Figure 1:**
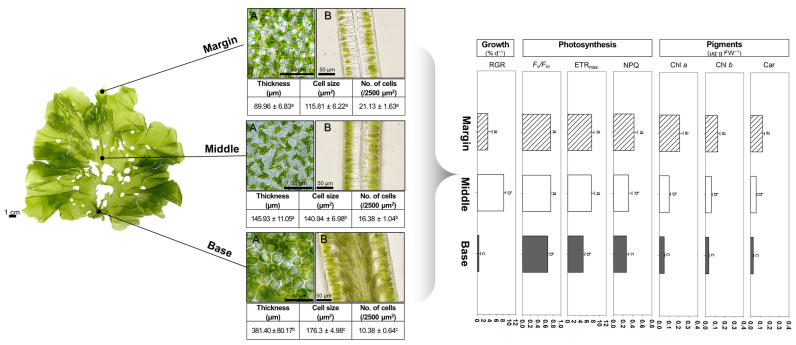
Comparison of morphological, physiological, and biochemical characteristics among margins, midsections, and basal sections in the thallus of *Ulva australis*. The left side of the figure depicts the organization of the thallus in *U. australis* as observed through a microscope, with a surface view (**A**) and a transverse section (**B**) of the thallus. On the right side, the figure shows the physiological and biochemical traits among the sections of *U. australis* thallus, including relative growth rate (RGR), maximum potential quantum efficiency of photosystem II (*F_v_*/*F_m_*), maximum electron transport rate (ETR_max_), non-photochemical quenching (NPQ), chlorophyll *a* (Chl *a*), chlorophyll *b* (Chl *b*), and carotenoids (Car). Data represent the mean values of eight replicates, and standard deviations are shown as error bars. Statistically significant differences at *p* < 0.05 (one-way ANOVA, LSD) are denoted using different letters.

In descending order, the thicknesses of the different sections were as follows: basal thallus (381.40 ± 80.17 µm); middle thallus (145.93 ± 11.05 µm); the marginal thallus (89.96 ± 6.83 µm). Cell size was also greatest in basal sections (176.3 ± 4.98 µm^2^), followed by middle (140.94 ± 6.98 µm^2^) and marginal thallus sections (115.81 ± 6.22 µm^2^). Furthermore, the number of cells was found to be inversely proportional to cell size: 21.13 ± 1.63 cells for the marginal, 16.38 ± 1.04 cells for the middle, and 10.38 ± 0.64 cells for the basal sections (Figure 1). 

Generally, *U. australis* is known to be morphologically simple, exhibiting little functional differentiation in its thalli. In addition, the growth points of *U. australis* have been shown to be randomly distributed across its thallus, causing an irregular expansion and controlled bilayer formation [4]. In contrast with these findings, our morphological measurements revealed significant differentiation across the different sections of the thallus. The findings linked to thallus thickness and cell size in this study are within the range of those reported by Han et al. [1] and, more generally for the genus, present a thickness and cell size ranging between 30–100 μm and 7–23 × 6–37 μm (length × width), respectively [30].

The relative growth rate (RGR) was greatest in the middle thallus (8.28 ± 0.64% d^−1^), followed by the marginal (3.35 ± 1.30% d^−1^) and basal thalli (0.51 ± 0.48% d^−1^) (Figure 1). The greater growth of the middle sections may be because *U. australis* can only reproduce from marginal thalli, which tend to initially grow rapidly and then stop in preparation for reproduction, as shown by Han et al. [1] and Park [26]. The slow growth of the basal sections of the thalli has been observed in a previous study [1]. The different RGRs observed between thallus sections in *U. australis* provide further evidence for functional differentiation along the different sections of the thallus, namely, the margins for reproduction, the middle for vegetative growth, and the base for anchoring and inter-annual carry-over.

Our assessment of photosynthetic capacity using two endpoints, *F*_v_/*F*_m_ and ETR_max_, showed that both were significantly higher in the marginal (0.73 ± 0.01 and 7.52 ± 0.70) and middle (0.74 ± 0.01 and 7.37 ± 0.95) thallus sections than in the basal sections (0.65 ± 0.01 and 4.86 ± 0.52) (Figure 1). The inter-sectional variation in photosynthetic efficiency may be linked to differences in cell size and morphology. Specifically, cell size and morphology have tended to be smallest and thinnest in the marginal thallus sections and largest and thickest in the basal thallus sections, respectively. This is in line with smaller cells having been reported to have a higher photosynthetic capacity [31,32,33]. Additionally, there is a relatively low packing effect, resulting in more efficient photon absorption in the pigments in thinner thalli [34,35].

Non-photochemical quenching (NPQ) approximates the ability of chloroplasts to dissipate excess excitation energy as heat. This process is useful for quantifying the resistance of plants to stress [36]. NPQ was highest in marginal thallus sections (0.43 ± 0.07), followed by the middle (0.31 ± 0.07) and basal thallus sections (0.27 ± 0.04) (Figure 1). In *Ulva* spp., the NPQ is highly correlated with the xanthophyll cycle; xanthophyll is one of the main constituents of carotenoids [37,38,39]. In this respect, it is noteworthy that the ranking of the NPQ values along the different thallus sections seems to be congruent with that of the carotenoid contents in the corresponding thallus sections.

Chlorophyll *a* content was greatest in the marginal thalli (0.21 ± 0.04 µg·g FW^−1^), followed by middle (0.10 ± 0.02 µg·g FW^−1^) and basal (0.05 ± 0.01 µg·g FW^−1^) thalli. Chl *b* and carotenoids were most abundant in the marginal thalli (0.13 ± 0.02 and 0.12 ± 0.02 µg·g FW^−1^), followed by the middle (0.06 ± 0.01 and 0.06 ± 0.01 µg·g FW^−1^) and basal thalli (0.03 ± 0.01 and 0.03 ± 0.004 μg·g FW^−1^) (Figure 1). The differences in the pigment concentrations of the different thallus sections could explain why *F*_v_/*F*_m_ and ETR_max_ were higher in the marginal thallus sections compared to those in the basal thallus sections.

### 3.2. Effects of Metals on Six Different Endpoints of Three Different Thallus Sections

Growth is an integrative measure of plant metabolism and is therefore likely to be sensitive to the adverse effects of environmental pollutants. EC_50_ values based on the RGR of *U. australis* exposed to metals varied across thallus sections and the specific metals (Table 2) used. Specifically, in the marginal and middle thallus sections, the RGR was most sensitive to Cu (EC_50_ values of 0.06 and 0.05 mg∙L^−1^, respectively) and least sensitive to Ni (EC_50_ > 0.5 mg∙L^−1^). The RGR of basal sections was most sensitive to Ni (EC_50_ = 0.03 mg∙L^−1^) and least sensitive to As (EC_50_ > 12.8 mg∙L^−1^) (Table 2). Only a few studies have been conducted on the effect of Cu on the growth of *U. australis*; however, Han et al. [15] found that growth was inhibited by 50% at 0.05–0.1 mg∙L^−1^ Cu. This value is similar to the EC_50_ for marginal and middle thallus sections obtained in this study. There is limited information on the effects of Ni toxicity on the physiology of *U. australis*. Han et al. [24] found that the spore release of *U. australis* was inhibited by 50% at a Ni concentration of 0.31 mg∙L^−1^, which is 10-fold greater (10-fold less sensitive) than the EC_50_ values for the basal thallus sections observed in this study. This is because high concentrations of Ni can induce oxidative stress in algae [40,41]. It is notable that the concentration of carotenoids was lower in basal thallus sections compared to in the middle and marginal sections. This may be due to the increased sensitivity to Ni in the basal thallus sections (Table 2), as carotenoids can act as free radical scavengers in algae [26], and high concentrations of carotenoids protect cells from oxidative stress [37].

The metal sensitivity of two photosynthetic endpoints, namely, maximum potential photosystem II quantum efficiency (*F_v_*/*F_m_*) and maximum electron transport rate (ETR_max_), also varied across specific metals and thallus sections. *F_v_*/*F_m_* in the marginal thallus sections was most sensitive to Ag (EC_50_ = 0.26 mg∙L^−1^) and least sensitive to As (>12.8 mg∙L^−1^), whereas ETR_max_ was most sensitive to Cu (0.08 mg∙L^−1^) and least sensitive to Cd (>8 mg∙L^−1^) (Table 2). In the middle thallus sections, the most toxic metal based on EC_50_ values for *F_v_*/*F_m_* was Cu (0.20 mg∙L^−1^), while the most toxic metal for ETR_max_ was Ag (0.06 mg∙L^−1^). In contrast, the least toxic for both endpoints (Table 2) was Cd (>8 mg∙L^−1^). In the basal part of the thallus, Ag was the most toxic, while Cd was the least toxic metal for both *F_v_*/*F_m_* (0.23 and >8 mg∙L^−1^) and ETR_max_ (0.07 and >8 mg∙L^−1^).

Most metals have an inhibitory effect on the metabolism of plants; however, certain metals are essential for photosynthesis. Nevertheless, these metals also become toxic to plants at high concentrations, negatively impacting photosynthesis [42]. For example, copper (Cu) is an important co-factor for plastocyanin and cytochrome oxidases, which are involved in key physiological processes in plants, such as photosynthesis and respiration [43]. However, excessive levels of Cu can adversely impact the photosynthetic electron transport chain and reduce photosynthetic efficiency [44]. Han et al. [15] found that *F_v_*/*F_m_* was significantly suppressed in the middle sections of the thalli of *U. australis* and *U. armoricana* at an exposure of 0.25 mg∙L^−1^. In *U. australis*, Kumar et al. [45] found EC_50_s of 0.37–0.48 mg∙L^−1^ Cu for *F_v_*/*F_m_* and 0.16–0.21 mg∙L^−1^ Cu for ETR_max_, which are in line with our findings (Table 3).

Silver is becoming an increasingly common industrial pollutant that is ionized in water and can bind to the active sites of photosynthetic enzymes and proteins in algae, damaging their photosynthetic system [40,41,42]. In *U. lactuca*, a 48-h exposure to 0.03 mg∙L^−1^ AgNO_3_ resulted in a reduction in chlorophyll *a* fluorescence by approximately 50% compared to the control [42]. This EC_50_ is similar to the Ag sensitivity of ETR_max_ for the basal and middle thallus sections in this study. Within the context of Ag exposure, the effective quantum yield (*F*′*_v_*/*F*′*_m_*) in the middle section of *U. lactuca* significantly decreased in exposure to 0.02 mg∙L^−1^; however, it did not decrease by more than 50%, even when exposed to 0.14 mg∙L^−1^ [42].

Metals directly affect chlorophyll pigments and, thus, interfere with photosynthesis and plant metabolism. Cu was the most toxic for pigments in marginal thallus, whereas Ag was most toxic for those in the middle and basal thalli. The least toxic metal for pigments in all thallus sections was As (Table 2). Cu deactivates enzymes and proteins involved in photo-activity and deforms thylakoid membranes [46]. Cu may also impair the structure and function of chlorophyll by removing magnesium from both the antenna complex and the reaction centre [43]. Han et al. [15] calculated the EC_50_ values of photosynthetic pigments in *U. armoricana* and *U. australis* exposed to Cu for 72 h to be 0.25 mg∙L^−1^. This is similar to the EC_50_ of the middle thallus sections in this study (0.21 ± 0.03 mg∙L^−1^) (Table 3).

Ag damages the chloroplast microstructure and chlorophyll content in algae [47]. However, studies on the effects of Ag on the pigment content of *U. australis* are scarce.

**Table 3:** List of six of metal toxicity using different thallus sections of *Ulva australis*.

Endpoints	Metals	Thallus Sections	Test Period (h)	EC50 (mg∙L−1)	CV (%)	References
Growth (RGR)	Ag	Margin	72	0.08	18.67	This study
		Middle	72	0.05	14.38	This study
		Base	72	>0.32	-	This study
	As	Margin	72	2.35	28.20	This study
		Middle	72	0.98	19.37	This study
		Base	72	>12.8	-	This study
	Cd	Margin	72	6.21	3.79	This study
		Middle	72	1.89	12.09	This study
		Base	72	2.88	16.33	This study
	Cr	Margin	72	3.94	32.44	This study
		Middle	72	0.87	28.72	This study
		Base	72	0.74	46.47	This study
	Cu	Margin	72	0.06	13.22	This study
		Middle	72	0.05	24.38	This study
			72	0.05–0.1, ca.	-	Han et al. [15]
		Base	72	0.12	4.04	This study
	Ni	Margin	72	>0.5	-	This study
		Middle	72	>0.5	-	This study
		Base	72	0.03	35.56	This study
*F_v_*/*F_m_*	Ag	Margin	72	0.26	2.71	This study
		Middle	72	0.30	-	This study
		Base	72	0.23	2.41	This study
	As	Margin	72	>12.8	-	This study
		Middle	72	5.03	0.79	This study
		Base	72	6.75	9.96	This study
	Cd	Margin	72	>8	-	This study
		Middle	72	>8	-	This study
		Base	72	>8	-	This study
	Cr	Margin	72	>6.4	-	This study
		Middle	72	4.89	7.85	This study
		Base	72	>6.4	-	This study
	Cu	Margin	72	>1.2	-	This study
		Middle	72	0.20	23.36	This study
			24	0.48	6.04	Kumar et al. [45]
			48	0.37	5.5	Kumar et al. [45]
			72	0.25, ca.	-	Han et al. [15]
			72	0.35	5.41	Kumar et al. [45]
			96	0.71	-	Kumar et al. [45]
		Base	72	0.67	8.37	This study
	Ni	Margin	72	>0.5	-	This study
		Middle	72	>0.5	-	This study
		Base	72	>0.5	-	This study
ETR_max_	Ag	Margin	72	0.23	1.80	This study
		Middle	72	0.06	15.27	This study
		Base	72	0.07	25.14	This study
	As	Margin	72	5.19	29.05	This study
		Middle	72	3.01	10.70	This study
		Base	72	4.99	4.82	This study
	Cd	Margin	72	>8	-	This study
		Middle	72	>8	-	This study
		Base	72	>8	-	This study
	Cr	Margin	72	0.22	6.36	This study
		Middle	72	1.29	23.56	This study
		Base	72	2.22	28.00	This study
	Cu	Margin	72	0.08	31.47	This study
		Middle	72	0.14	15.05	This study
			24	0.21	8.98	Kumar et al. [45]
			48	0.16	-	Kumar et al. [45]
			72	0.05–0.10, ca.	-	Han et al. [15]
			72	0.29	11.80	Kumar et al. [45]
			96	0.36	2.60	Kumar et al. [45]
		Base	72	0.11	15.68	This study
	Ni	Margin	72	>0.5	-	This study
		Middle	72	>0.5	-	This study
		Base	72	>0.5	-	This study
Chl *a*	Ag	Margin	72	0.22	4.19	This study
		Middle	72	0.11	4.56	This study
		Base	72	0.12	26.01	This study
	As	Margin	72	>12.8	-	This study
		Middle	72	>12.8	-	This study
		Base	72	>12.8	-	This study
	Cd	Margin	72	>8	-	This study
		Middle	72	>8	-	This study
		Base	72	>8	-	This study
	Cr	Margin	72	5.71	-	This study
		Middle	72	4.11	13.08	This study
		Base	72	>6.4	-	This study
	Cu	Margin	72	0.05	10.39	This study
		Middle	72	0.18	17.85	This study
			72	0.25, ca.	-	Han et al. [15]
		Base	72	0.92	10.89	This study
	Ni	Margin	72	>0.5	-	This study
		Middle	72	>0.5	-	This study
		Base	72	>0.5	-	This study
Chl *b*	Ag	Margin	72	0.22	5.11	This study
		Middle	72	0.12	3.78	This study
		Base	72	0.10	40.28	This study
	As	Margin	72	>12.8	-	This study
		Middle	72	>12.8	-	This study
		Base	72	>12.8	-	This study
	Cd	Margin	72	>8	-	This study
		Middle	72	>8	-	This study
		Base	72	>8	-	This study
	Cr	Margin	72	6.04	-	This study
		Middle	72	4.55	11.06	This study
		Base	72	>6.4	-	This study
	Cu	Margin	72	0.06	7.77	This study
		Middle	72	0.24	11.56	This study
		Base	72	>1.2	-	This study
	Ni	Margin	72	>0.5	-	This study
		Middle	72	>0.5	-	This study
		Base	72	>0.5	-	This study
Carotenoid	Ag	Margin	72	0.20	9.74	This study
		Middle	72	0.12	3.87	This study
		Base	72	0.11	34.41	This study
	As	Margin	72	>12.8	-	This study
		Middle	72	>12.8	-	This study
		Base	72	>12.8	-	This study
	Cd	Margin	72	>8	-	This study
		Middle	72	>8	-	This study
		Base	72	>8	-	This study
	Cr	Margin	72	5.20	-	This study
		Middle	72	4.48	10.83	This study
		Base	72	>6.4	-	This study
	Cu	Margin	72	0.05	11.00	This study
		Middle	72	0.21	9.81	This study
		Base	72	1.09	-	This study
	Ni	Margin	72	>0.5	-	This study
		Middle	72	>0.5	-	This study
		Base	72	>0.5	-	This study
Antioxidation	Cu	Middle	72	0.1–0.25, ca.	-	Han et al. [15]
Gametophyte (length)	Cd	Margin	144	0.19	-	Han et al. [48]
	Cu	Margin	144	0.02	6.41	Han et al. [48]
Gametophyte (No. of cells)	Cd	Margin	144	0.20	-	Han et al. [48]
	Cu	Margin	144	0.03	8.32	Han et al. [48]
Reproduction	Ag	Margin	96	0.13	11.41	Lee et al. [16]
	Cd	Margin	72–120	0.22	-	Han et al. [23]
	Cd	Margin	96	0.72	17.10	Lee et al. [20]
	Cu	Margin	72–120	0.06	-	Han et al. [23]
	Cu	Margin	96	0.12	9.67	Lee et al. [20]
Sporulation (visual)	Ag	Margin	96	0.05	3.11	Han et al. [24]
	As	Margin	96	1.03	10.54	Han et al. [24]
	Cd	Margin	96	0.27	7.63	Han et al. [24]
	Cr	Margin	96	1.50	2.08	Han et al. [24]
	Cu	Margin	96	0.10	2.46	Han et al. [24]
	Ni	Margin	96	0.98	7.30	Han et al. [24]
Sporulation (image analyser)	Ag	Margin	96	0.05	2.95	Han et al. [24]
	As	Margin	96	0.86	10.48	Han et al. [24]
	Cd	Margin	96	0.26	8.38	Han et al. [24]
	Cr	Margin	96	1.45	2.90	Han et al. [24]
	Cu	Margin	96	0.10	3.01	Han et al. [24]
	Ni	Margin	96	0.95	4.60	Han et al. [24]
Sporulation	Cd	Margin	120	0.33	10.43, ca.	Han and Choi [22]
	Cu	Margin	120	0.06	32.78, ca.	Han and Choi [22]
Spore release	Ag	Margin	96	0.04	2.53	Han et al. [24]
	As	Margin	96	0.45	19.56	Han et al. [24]
	Cd	Margin	96	0.10	11.71	Han et al. [19]
		Margin	96	0.22	6.55	Han et al. [24]
		Margin	96	0.26	8.38	Oh et al. [49]
	Cr	Margin	96	0.80	4.26	Han et al. [24]
	Cu	Margin	96	0.04	8.40	Han et al. [19]
		Margin	96	0.08	5.02	Han et al. [24]
		Margin	96	0.10	3.01	Oh et al. [49]
	Ni	Margin	96	0.31	4.13	Han et al. [24]
Spore germination	Cd	Margin	72	0.79	3.29	Han et al. [48]
	Cu	Margin	72	0.02	3.39	Han et al. [48]

### 3.3. Effect of Metals on rbcL Expression

**Figure 2:**
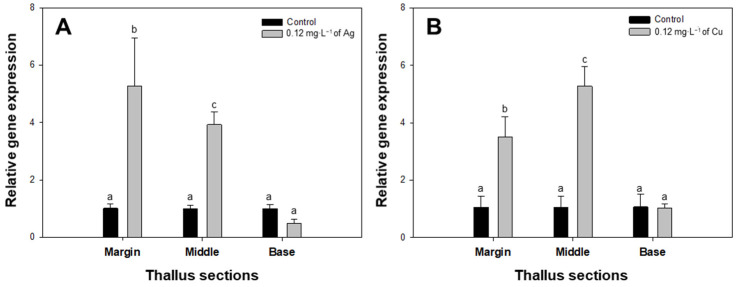
Relative *rbcL* expression in different thallus sections of *Ulva australis* under Ag and Cu exposure. Each thallus was exposed to 0.12 mg∙L^−1^ of Ag (**A**) and Cu (**B**). Data are the means of four replicates (± 95% confidence intervals). Different letters indicate statistically significant differences at *p* < 0.05 (one-way ANOVA, LSD).

After exposure to 0.12 mg∙L^−1^ of Ag or Cu, the expression of *rbcL* increased across the middle and marginal sections of the thallus of *U. australis*, but not in the basal sections (Figure 2). Upon exposure to Ag, the expression level of *rbcL* increased five-fold (from 1.01 ± 0.14 to 5.28 ± 1.48) and four-fold (from 1.01 ± 0.11 to 3.93 ± 0.38) in the middle and marginal thallus sections, respectively, relative to the control (Figure 2). Gene expression in response to Cu increased three-fold in the marginal thallus (from 1.05 ± 0.33 to 3.50 ± 0.62) and four-fold in the middle thallus (from 1.06 ± 0.34 to 5.27 ± 0.61) (Figure 2). Conversely, the *rbcL* expression levels in basal sections were not significantly different from those of the control group.

Our study showed that the expression of the *rbcL* gene increased in the marginal and middle sections of the thalli exposed to metal toxicity. This up-regulation of gene transcription may represent a response from *U*. *australis* to these phytotoxic substances to ensure photosynthesis and survival. However, this increase could lead to more severe oxidative damage via increased ROS production [44]. Further studies are required to confirm this hypothesis. The levels of 18S rRNA can be dynamic in response to stress; therefore, the inclusion of the transcription levels of a reference gene from the *U. australis* genome could help isolate the effects of metal toxicity, specifically on Rubisco-related gene expression.

### 3.4. Sensitivity and Reliability of the Endpoints of the Three Different Thallus Sections

**Figure 3:**
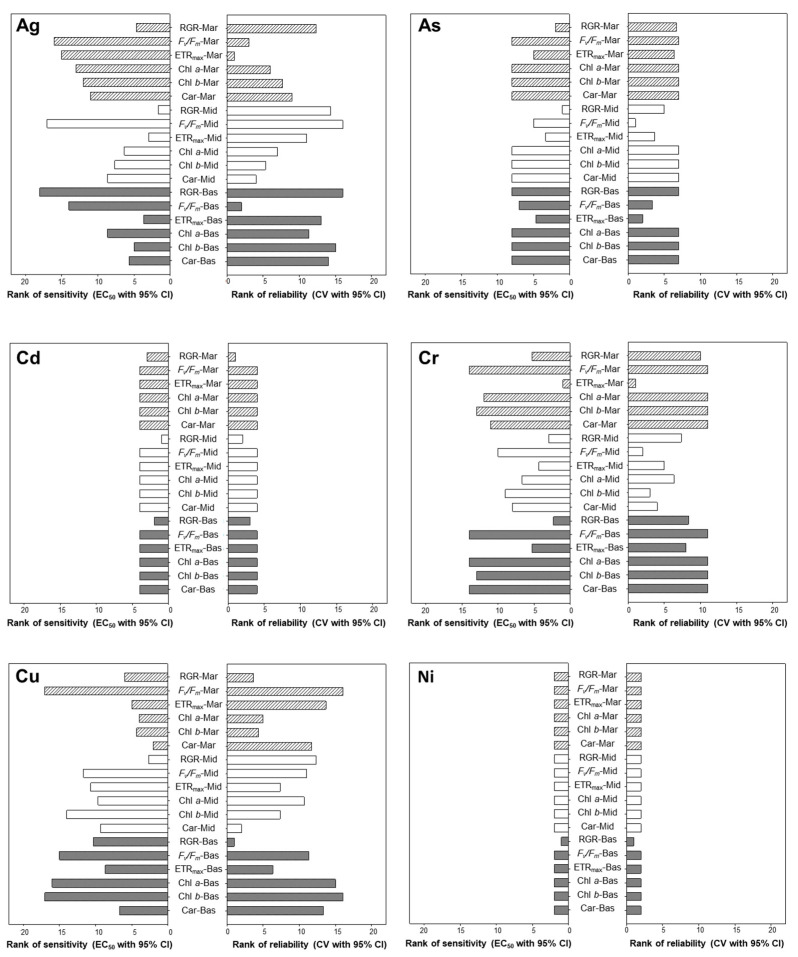
Rank of sensitivity and reliability of various endpoints for each metal species (i.e., Ag, As, Cd, Cr, Cu, and Ni). The lower the EC_50_ value was, the more sensitive the endpoint; moreover, the lower the coefficient of variation (CV) was, the more reliable the endpoint. Mean ranks were calculated for the endpoints, thallus sections, and metals independently using sensitivity and reliability. RGR, Relative growth rate; *F_v_*/*F_m_*, Maximum potential quantum efficiency of photosystem II; ETR_max_, Maximum electron transport rate; Chl *a*, Chlorophyll *a*; Chl *b*, Chlorophyll *b*; Car, Carotenoid; Mar, Marginal region of *Ulva australis* thallus; Mid, Middle section of *U. australis* thallus; Bas, Basal section of *U. australis* thallus.

Figure 3 shows the pairs of the most sensitive and reliable endpoints for each metal species. For Ag, the RGR of the marginal thallus section and the ETR_max_ of the middle and basal thallus sections were both found to be highly sensitive and reliable. Among them, the ETR_max_ of the mid-section was found to be the most sensitive and reliable endpoint for the diagnosis of Ag toxicity.

With regard to As, the RGR of the marginal and middle thallus sections and the ETR_max_ of the basal thallus section were found to be highly sensitive and reliable. Among these, the RGR of the mid-thallus was found to be the most sensitive and reliable endpoint for the diagnosis of As toxicity.

For Cd, the RGR was found to be highly sensitive and reliable for all three different thallus sections. Among them, the RGR of the middle section was found to be the most sensitive and reliable endpoint for the diagnosis of Cd toxicity.

For Cr, the ETR_max_ of the marginal thallus section and the RGR of the middle and basal thallus sections were found to be highly sensitive and reliable. Among them, the ETR_max_ of the marginal section was found to be the most sensitive and reliable endpoint for the diagnosis of Cr toxicity.

For Cu, it was found that the Chl *b* of the marginal thallus section and the RGR of the middle thallus section and the ETR_max_ of the basal thallus section were highly sensitive and reliable. Among them, marginal Chl *b* was found to be the most sensitive and reliable endpoint for the diagnosis of Cu toxicity.

For Ni, the RGR of the basal section was found to be the most sensitive and reliable endpoint for the diagnosis of Ni toxicity.

## 4. Conclusions

Morphological, physiological, and biochemical differences were characterised across different sections of the thallus of *U. australis*. The resultant trends suggest that the three different sections of the thallus are differentiated across functional axes. Specifically, marginal sections of the thallus have reproductive functions (i.e., spore formation and release), the middle sections are involved in vegetative growth, and, finally, basal sections of the thallus anchor algae support growth in subsequent growth cycles. Moreover, each thallus section responded uniquely upon exposure to the six metals (i.e., Ag, As, Cd, Cu, Cr, and Ni). Ag and Cu were consistently the most toxic metals across all the tested endpoints. Based on these results, we propose the following endpoints as the most appropriate for evaluating the toxicity of the six metals we tested: ETR_max_ of the middle thallus for Ag toxicity; RGR of the middle thallus for As and Cd; ETR_max_ of the marginal thallus for Cr; Chl *b* of the marginal thallus for Cu; RGR of the basal thallus for Ni.

Our study suggests that *U. australis*-based endpoints are generally suitable for evaluating pollution given the quality standards for wastewater discharge (QSWD) in Korea and the US. The Korean Ministry of Environment currently has no standard limit for Ag discharges but has set 1.0 mg∙L^−1^ for Cu discharge. In the US, the permissible limits for Ag and Cu are 0.5 mg∙L^−1^ and 0.25 mg∙L^−1^, respectively (USEPA). The EC_50_ values for the inhibition of ETR_max_ in the middle (0.06 mg∙L^−1^) and Chl *b* in the marginal thallus sections (0.06 mg∙L^−1^) were all lower than the QSWD values of Ag and Cu in Korea and the US. Moreover, the majority of EC_50_ values obtained from multiple endpoints in thallus sections exposed to Ag and Cu were lower than the QSWD values in Korea and the US. Notably, the RGR (0.03 mg∙L^−1^) of the basal thallus sections was sufficiently sensitive enough to detect the presence of Ni at concentrations exceeding the permissible values. Therefore, the EC_50_ values obtained from the bioassays could be used to establish ecologically sound and acceptable standards for wastewater discharge.

It is also important to note that the permitted levels of Ag and Cu in the effluent are higher than the safety limits for the two endpoints (i.e., ETR_max_ of the middle thallus section and Chl *b* of the marginal thallus section), highlighting the need to review the current management settings for Ag and Cu in wastewater if *U. australis* is to survive as a primary producer, providing energy, food, and nursery grounds for organisms of higher trophic levels in marine aquatic ecosystems.

In conclusion, our study showed that different sections of the thallus of *U. australis* can be used independently to assess wastewater discharge due to their different responses to different pollutants. Specifically, each thallus section provides unique endpoints that are similar to multi-species assays in detecting the inference of a specific metal toxicant, thereby providing valuable information on the level of pollution in a given environment.

There has been increasing interest in the use of multi-species tests for ecotoxicity assessment because of their greater ecological relevance compared to single-species tests. However, there are several challenges to conducting multi-species tests, including the difficulty in drawing conclusions when there are differences in sensitivity between test organisms from different trophic levels, the increased variation within organisms due to seasonal adaptation, and the challenges and costs associated with cultivation and maintenance.

On the other hand, *U. australis* has different characteristics depending on the section of its thallus. The marginal thallus is suitable for reproduction, the central part promotes growth, and the basal part serves as an attachment site. Therefore, by developing an ecotoxicity assessment method that incorporates different endpoints for each specific section of the *U. australis* thallus, it will be possible to overcome the challenges inherent in the development of multi-species test methods while addressing the limitations of single-species test methods. Here, we propose that the present *U. australis* method does not replace multi-species tests, but rather serves as a complementary approach to overcome their limitations. This finding is significant since it offers a more cost-effective and time-efficient method of assessing metal toxicity, which is crucial for environmental protection and public health. By using multiple endpoints (i.e., that correspond to specific sections of the thallus), this approach can facilitate a comprehensible and dependable assessment of the risks posed by metal pollution to aquatic ecosystems.

## Figures and Tables

**Table 1:** Final concentration ranges for testing toxicity of each metal across different thallus sections of *Ulva australis*.

Metals	Chemical Formula	Concentrations (mg∙L^−1^)	CAS no.	Manufacturer
Cu	CuSO_4_	0.0375–1.2	7758-98-7	Showa, Japan
Cr	K_2_Cr_2_O_7_	0.4–6.4	7778-50-9	Showa, Japan
Ni	NiSO_4_(NH_4_)_2_SO_4_	0.03125–0.5	15699-18-0	Showa, Japan
As	As_2_O_3_	0.8–12.8	1327-53-3	Showa, Japan
Cd	CdSO_4_	0.5–8	10124-36-4	Showa, Japan
Ag	AgNO_3_	0.01–0.32	7761-88-8	Showa, Japan

**Table 2:** EC_50_ values (with 95% CI; mg∙L^−1^) for various endpoints in each thallus section exposed to six metals.

Endpoints	Thallus Sections	Metals					
		Ag	As	Cd	Cr	Cu	Ni
RGR	Margin	0.08 (0.06–0.11)	2.35 (1.29–3.70)	6.21 (5.71–6.55)	3.94 (0.38–4.96)	0.06 (0.05–0.08)	>0.5
	Middle	0.05 (0.04–0.07)	0.98 (0.65–1.34)	1.89 (1.59–2.57)	0.87 (0.38–1.37)	0.05 (0.03–0.07)	>0.5
	Base	>0.32	>12.8	2.88 (1.74–3.27)	0.74 (0.27–1.68)	0.12 (0.11–0.13)	0.03 (0.02–0.05)
*F_v_*/*F_m_*	Margin	0.26 (0.25–0.28)	>12.8	>8	>6.4	>1.2	>0.5
	Middle	0.30	5.03 (4.96–5.11)	>8	4.89 (4.26–5.73)	0.20 (0.07–0.25)	>0.5
	Base	0.23 (0.21–0.24)	6.75 (5.96–8.20)	>8	>6.4	0.67 (0.52–0.75)	>0.5
ETR_max_	Margin	0.23 (0.219–0.236)	5.19 (1.96–7.46)	>8	0.22 (0.21–0.26)	0.08 (0.03–0.11)	>0.5
	Middle	0.06 (0.041–0.076)	3.01 (2.55–3.75)	>8	1.29 (0.74–1.83)	0.14 (0.07–0.17)	>0.5
	Base	0.07 (0.04–0.11)	4.99 (4.62–5.57)	>8	2.22 (0.98–3.34)	0.11 (0.07–0.13)	>0.5
Chl *a*	Margin	0.22 (0.19–0.23)	>12.8	>8	5.71	0.05 (0.04–0.07)	>0.5
	Middle	0.11 (0.10–0.12)	>12.8	>8	4.11 (2.53–4.92)	0.18 (0.04–0.22)	>0.5
	Base	0.12 (0.05–0.21)	>12.8	>8	>6.4	0.92 (0.56–1.08)	>0.5
Chl *b*	Margin	0.22 (0.17–0.23)	>12.8	>8	6.04	0.06 (0.05–0.07)	>0.5
	Middle	0.12 (0.10–0.13)	>12.8	>8	4.55 (2.87–5.41)	0.24 (0.17–0.32)	>0.5
	Base	0.10 (0.01–0.21)	>12.8	>8	>6.4	>1.2	>0.5
Car	Margin	0.20 (0.16–0.22)	>12.8	>8	5.20	0.05 (0.03–0.06)	>0.5
	Middle	0.12 (0.11–0.13)	>12.8	>8	4.48 (2.68–5.27)	0.21 (0.16–0.25)	>0.5
	Base	0.11 (0.02–0.20)	>12.8	>8	>6.4	1.09	>0.5

## Data Availability

The data presented in this study are available on request from the corresponding author.
